# Effects of cannabis on visual function and self-perceived visual quality

**DOI:** 10.1038/s41598-021-81070-5

**Published:** 2021-01-18

**Authors:** Sonia Ortiz-Peregrina, Carolina Ortiz, Miriam Casares-López, José R. Jiménez, Rosario G. Anera

**Affiliations:** Department of Optics, Laboratory of Vision Sciences and Applications, University of Granada, Edificio Mecenas, Av. Fuentenueva s/n, 18071 Granada, Spain

**Keywords:** Medical research, Risk factors

## Abstract

Cannabis is one of the most used drugs of abuse in the world. The objective of this study was to analyze the effects of smoking cannabis on vision and to relate these to those perceived by the user. Thirty-one cannabis users participated in this study. Visual function assessment was carried out in a baseline session as well as after smoking cannabis. We evaluated static visual acuity, contrast sensitivity, stereoacuity, accommodative response, straylight, night-vision disturbances (halos) and pupil size. The participants were also divided into two groups depending on whether they perceived their vision to have worsened after smoking cannabis. A logistic regression analysis was employed to identify which visual test could best predict self-perceived visual effects. The study found that smoking cannabis has significant adverse effects on all the visual parameters analyzed (*p* < 0.05). Self-perceived visual quality results revealed that about two thirds of the sample think that smoking cannabis impairs their vision. Contrast sensitivity, specifically for the spatial frequency 18 cpd, was identified as the only visual parameter significantly associated with self-perceived visual quality (Odds Ratio: 1.135; *p* = 0.040). Smoking cannabis is associated with negative effects on visual function. Self-perceived visual quality after smoking cannabis could be related to impaired contrast sensitivity.

## Introduction

Cannabis is one of the most widely used drugs of abuse in the world, and the consequences of this involve increasing health concerns. Data from the European Monitoring Centre for Drugs and Drug Addiction (2019) shows that around 15% of young Europeans, aged between 15 and 34, smoked cannabis in 2018^1^. Spain has one of the highest incidences of cannabis use and the prevalence of consumption has increased in recent years. The survey on alcohol and drugs in Spain (EDADES) (2017) showed that in 2017, 35% of the population aged 15–64 had used cannabis at some time^2^. In addition, use of this drug is expected to increase over the next few years, prompted by its recent legalization for medical and recreational use in several countries, as well as the introduction of a legal cannabis industry^3^.

Regular use of this drug, of which the main psychoactive component is ∆9-tetrahydrocannabinol (THC), has been associated with psychiatric disorders. These include outcomes such as schizophrenia^4,5^ and impaired memory, attention, and psychomotor coordination^6–8^. These problems are associated with the presence of cannabinoid receptors in the central nervous system^9^. However, despite the fact that cannabis use causes sensory distortion and that there are also cannabinoid receptors located within the human visual system, the effects on visual physiology have been little explored^9,10^. Some decades ago certain studies addressed the effects of cannabis intoxication on visual perception^11,12^, but without explicitly controlling for confounding factors such as concomitant use of alcohol or tobacco, or the exact quantity consumed. Their results indicating a deterioration in color vision^11^, in static and dynamic visual acuity^13,14^, in adaptation to darkness^14^, and a longer glare-recovery time^12^. Cannabis intoxication also seems to impair visual processing, with effects including a reduction in the binocular depth inversion illusion, which occurs when a three-dimensional object is presented pseudoscopically^15^. This effect is also noted in long-term users^16^, as are other effects related to a slowing down of visual information processing, such as dysfunction in the ganglion cells^17^, reduced contrast sensitivity^18,19^, impaired motion perception^19,20^ and a drop in the quality of reading-related ocular movements^21^. In contrast, other observation-based or case study work has argued that acute cannabis intoxication exerts positive effects on nighttime vision, with improved adaptation to darkness and scotopic sensitivity^22,23^. Work on the effects smoking cannabis has on the ocular tissues have produced conflicting results with regard to pupil size. However, it has been demonstrated that there is reduced ocular sensitivity and intraocular pressure (IOP), conjunctival vasodilation and increased tear production^24^.

Surprisingly, despite all these consequences and the fact that it is an illegal drug, it is quite common to find misperceptions of the cannabis-use-associated risks, which could lead to an ever-increasing number of users. The results of a US national survey conducted from 2002 to 2014 revealed that the proportion of adults who think the risk from using cannabis 1–2 times a week is at least moderate decreased from 50.4 to 33.3% during that period^25^. This indicates that cannabis is perceived as being a “safe” drug^26^, an idea generated by erroneous beliefs about this substance, for instance, thinking that cannabis has positive effects on the brain, increasing creativity and helping treat mental illnesses^27^. In the case of vision, there is also misperception of the risk cannabis use presents. For this reason, a recent study asked heavy and light cannabis users, in addition to non-users, to rate their eyesight quality on a 5-point scale from excellent (1) to poor (5). The results obtained showed that heavy smokers perceived their quality of vision to be the same as that of the light users and those who had never used cannabis^28^.

As indicated above, some of the evidence about the effects of cannabis consumption on vision are inconclusive, especially with regard to night vision parameters. Moreover, there is a lack of data about the effects this drug has on important aspects of visual function such as stereoacuity (i.e. depth perception) or accommodative response, which are very important for the adequate performance of everyday tasks. Furthermore, while previous findings suggest that cannabis users rate their general quality of vision on a par with that of nonusers, they may experience certain effects during acute cannabis intoxication. This is important because the perceived effect could limit their willingness to carry out day-to-day tasks. We hypothesize that smoking cannabis could alter specific aspects of visual function such as visual acuity, stereoacuity, accommodative response and night-vision performance, and that some of these changes could determine the subjective perception of visual quality under acute intoxication.

Therefore, the aim of this study was to examine the effects of the recreational use of cannabis on a wide range of parameters that characterize visual function, and to analyze whether users' subjective visual perception of the consequences of cannabis smoking is related to the objectively assessed changes.

## Methods

This study was conducted in line with the Declaration of Helsinki and was prospectively approved by the University of Granada Human Research Ethics Committee (921/CCEIH/2019). Prior to participating in the study, the subjects were verbally informed of the details and possible consequences of the study, and a signed informed consent was obtained from each participant.

The study included thirty-one volunteers (20 males and 11 females) ranging in age from 19 to 43 (mean (SD), 23.4 (5.1) years). All the subjects were occasional cannabis users, i.e., self-reported cannabis use of at least once but less than four times/week over the three months prior to the study^29,30^. Exclusion criteria were: any history of other drug use (used more than 5 times in their lifetime), nonnormal corrected vision, binocular problems, a history of previous or current medical disease and alcohol or cannabis use disorders. To evaluate this last requirement, all participants completed the revised Cannabis Use Disorders Identification Test (CUDIT-r)^31^ and the Alcohol Use Disorders Identification Test (AUDIT)^32^. The participants were asked to abstain from cannabis use in the four days preceding the study and refrain from alcohol intake for at least 24 h prior to testing. Data on their use profile and self-perceived risk were collected using questionnaires.

### Visual function assessment

Static visual acuity (VA) was measured binocularly (logMAR) at a working distance of 5.5 m with the Pola VistaVision Visual Chart System (DMD Med Tech srl. Torino, Italy). The same device was employed to evaluate binocular contrast sensitivity (CS) at the recommended distance of 2.5 m (decimal notation). The spatial frequencies tested were: 0.75, 1.5, 3, 6, 12, and 18 cycles per degree (cpd), using Gabor patch gratings with three possible orientations (vertical, left, or right). The subject had to indicate the orientation of the gratings, beginning with the lowest spatial frequency and the highest contrast value available. The contrast value was progressively reduced until the participant was unable to respond correctly. The last correct response was taken as the contrast threshold. The same procedure was repeated to evaluate the other spatial frequencies, in ascending order. The average background luminance level of the monitor was 60 cd/m^2^ and the test was performed in dim surroundings.

Stereoacuity is defined as the capacity to perceive depth according to retinal disparities^33^. For far vision, stereoacuity was evaluated using the polarized stereotest implemented in the VistaVision monitor at 5.5 m. The stereotest can measure disparities ranging from 300 to 10 arcsec using polarized vertical lines. For near vision (40 cm), we employed the Fly Stereo Acuity Test (Stereo Optical Co., Inc, Chicago, IL), a polarized-based test that contains a graded circle test, evaluating disparities from 400 to 20 arcsec. For both tests the subjects used polarized glasses.

Accommodative response (AR) is defined as the ability to change ocular power in response to a change in fixation distance and the accuracy to maintain a steady level of focus at a chosen fixation distance^34^. When the AR is lower than the accommodative demand, this is termed accommodative lag, whereas if the AR is higher than the accommodative demand, it is known as accommodative lead. The accommodative response was objectively measured using the Grand Seiko WAM-5500 open field autorefractor (Grand Seiko Co. Ltd., Hiroshima, Japan). The participants had to look at a 2 cm high-contrast (Michelson = 79%) black star target presented on a white background card. The subjects looked at the target binocularly through a 12.5 × 22 cm open-field beam-splitter, although the autorefractor only allows data to be recorded from one eye at a time. We took an initial measurement at a viewing distance of 6 m (baseline refraction value) and repeated this procedure nine times. Then, for the near testing condition, the target was displayed in two locations (corresponding to two accommodative demands): 40 cm (2.5D) and 20 cm (5D), with nine measurements being taken for each distance evaluated. At all times, the alignment of the subject with the fixation target was checked (positioned in the observer’s gaze midline) to ensure on-axis measurements, while they were leaning their forehead and chin against rests. All the refractive measurements were converted to the spherical equivalent (SE). The accuracy of the accommodative response was determined according to the criteria of Poltavski et al., by subtracting the mean point of focus of the different measures and the baseline refraction value from the accommodative demand required by the target distance (in our case 2.5D and 5D)^35^.

Intraocular straylight is defined as the visual effect of light scattering in the optical eye media^36^. This causes a veiling luminance over the whole retina (a veil of straylight), reducing the contrast of the retinal image and causing disability glare^37^. To quantify the level of intraocular straylight we employed the C-Quant straylight meter (Oculus Optikgeräte GmbH, Germany), using the compensation comparison method^38,39^. In brief, the subjects had to compare the flickering of the two halves of a test field and indicate which was flickering the most strongly. Intraocular straylight was expressed as log(s), with higher values indicating more straylight and greater glare sensitivity. The measurements were taken monocularly and in a darkened room.

The Halo v1.0 test was used to evaluate the subject’s ability to detect peripheral stimuli in the presence of visual disturbances such as glare, halos or veils of straylight generated by a central high-luminance stimulus under low light conditions. This test allows the halos perceived by the observer to be quantified using the visual-disturbance index (VDI). This index has a value of between 0 and 1 (the higher the VDI, the greater the influence of halos and, therefore, the greater the difficulty in detecting peripheral stimuli). The subject’s task was to press the left button on a mouse whenever they detected a peripheral stimulus around a central luminous stimulus. The software also generates a graph of the results showing areas where the peripheral stimuli were either detected (green) or not detected (red) by the observer, delimiting the shape of the halo observed^40^.

All the visual tests were performed using the best optical correction and under natural pupil conditions. The visual function assessment was carried out in two randomized sessions (a baseline session involving no cannabis use and another after smoking the substance), with a washout period of approximately 7 days between these. We simulated the recreational use of cannabis, wherein participants prepared a cannabis cigarette as they would normally do for their habitual consumption, and they smoked it within about 10 min. The session involving cannabis use was conducted with a lag of no more than 20 min between the end of use and the beginning of the session. This time lapse after cannabis consumption was established because past research indicates that although THC plasma levels peak immediately after smoking, behavioral impairment occurs once plasma levels have dropped^41^. The testing sessions lasted about 75 min, guaranteeing a considerable psychoactive effect during the session after smoking cannabis given that this tapers off within 2–3 h^41^. Also, the order of the tests in each session was random. To obtain objective confirmation of cannabis use, a saliva drug test was performed using the Dräger DrugTest 5000 (Dräger Safety AG & Co. KGaA. Lübeck, Germany). This device also allowed us to ensure that no other substances had been used, including amphetamines, benzodiazepines, cocaine, methamphetamines, opiates, methadone, or ketamine. To check that the participants had not consumed alcohol, we measured the breath alcohol content (BrAC) with the Dräger Alcotest 7110 MK-III (Dräger Safety AG & Co. KGaA. Lübeck, Germany). Pupil size was recorded using the NeurOptics VIP-300 pupillometer (NeurOptics, Irvine, CA, USA). This parameter was measured at different background illuminations, simulating scotopic (background off), low mesopic (0.3 lx) and high mesopic (3 lx) viewing conditions. Additionally, all the participants were asked to complete a questionnaire on self-perceived visual quality after using cannabis on a 4-point scale. The possible responses were: 1 (much worse), 2 (slightly worse), 3 (no change), and 4 (improved).

### Data analysis

The data distribution normality was evaluated with the Kolmogorov–Smirnov test. Descriptive statistics were employed for visual parameters and questionnaire responses. The paired sample t-test was used to compare the baseline and post-cannabis-use sessions and, when normal distribution of data could not be assumed, we employed the Wilcoxon signed-rank test. The visual test results were also compared between sexes after smoking cannabis, employing an unpaired t-test or the Mann–Whitney U test for nonnormal distributions. The sample was then categorized into two groups according to the participants’ subjective perception of visual quality after cannabis use and compared by means of the unpaired t-test or Mann–Whitney U test when normality could not be assumed. The variables used for the comparisons were the mean differences between the conditions, which were calculated by subtracting the value obtained after smoking cannabis from the value obtained in the baseline session, for each visual parameter. Therefore, a negative value in the mean differences for visual acuity, stereoacuity, VDI and log(s) indicates that smoking cannabis worsens these visual parameters; however, for contrast sensitivity, a positive value indicates that smoking cannabis has a negative effect. A positive value in the mean differences for accommodative response indicates that cannabis use produces a (greater) accommodative lag. Lastly, and once the sample had been divided according to their opinions based on their own experiences, a binary logistic regression model was used to investigate whether any visual parameters were able to predict self-perceived visual quality after smoking cannabis, taking into account demographic factors such as age, sex, and the AUDIT and CUDIT-r scores. The parsimonious model was obtained by applying a stepwise backward elimination technique (5% significance level) and the maximum likelihood estimation. Statistical significance was set at *p* < 0.05.

## Results

**Figure 1:**
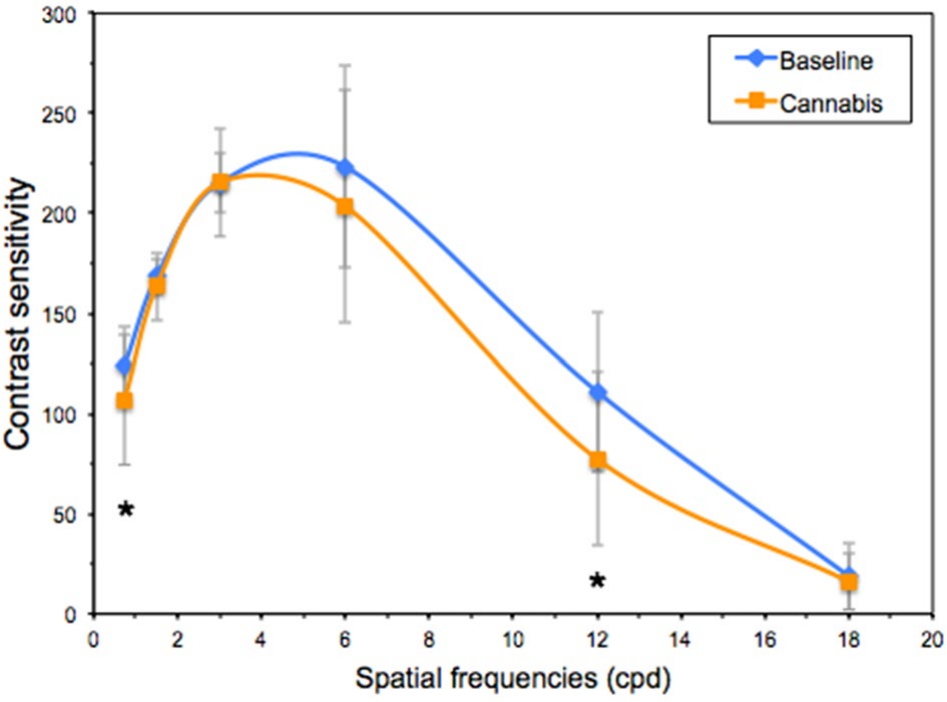
Mean binocular contrast-sensitivity function for the baseline session and after smoking cannabis. Error bars indicate the SD and * is used when *p* < 0.05 (Wilcoxon-signed ranks test). cpd, cycles per degree.

The paired t-test (or Wilcoxon test) indicated lower binocular visual acuity and poorer mean contrast sensitivity after smoking cannabis, with this deterioration being significant at the spatial frequencies 0.75 cpd (z = − 2.724; *p* = 0.006) and 12 cpd (z = − 3.234; *p* = 0.001) (Table 1, Fig. 1).

**Table 1:** Group mean and SD for visual parameters in the two sessions (baseline and after smoking cannabis).

	Baseline (mean ± SD)	Cannabis (mean (SD))	t/z	*p* value
Binocular VA (logMAR)	− 0.11 ± 0.05	− 0.06 ± 0.06	− 6.103	< 0.001
Binocular CS	143.43 ± 15.85	130.50 ± 19.49	4.439	< 0.001
Stereoacuity (arcsec) (5.5 m)^a^	50.67 ± 47.92	158.67 ± 104.64	− 4.461	< 0.001
Stereoacuity (arcsec) (0.4 m)^a^	22.23 ± 10.11	33.50 ± 28.76	− 3.528	< 0.001
Accommodative response (lag) (D) (0.4 m)	− 0.97 ± 0.50	− 1.22 ± 0.60	3.026	0.006
Accommodative response (lag) (D) (0.2 m)	− 1.28 ± 0.63	− 1.73 ± 0.77	3.892	0.001
Straylight (Log(s))^a^	0.85 ± 0.09	0.93 ± 0.15	− 3.573	< 0.001
Binocular VDI^a^	0.13 ± 0.05	0.15 ± 0.06	− 2.415	0.016
Pupil size (mm)				
Scotopic	6.71 ± 0.84	6.36 ± 0.66	4.046	< 0.001
Low mesopic	6.00 ± 0.87	6.03 ± 0.70	− 0.366	0.72
High mesopic	5.56 ± 0.89	5.59 ± 0.87	− 0.257	0.80

VA, visual acuity; CS, contrast sensitivity; VDI, visual disturbance index.

^a^Wilcoxon signed ranks test.

**Figure 2:**
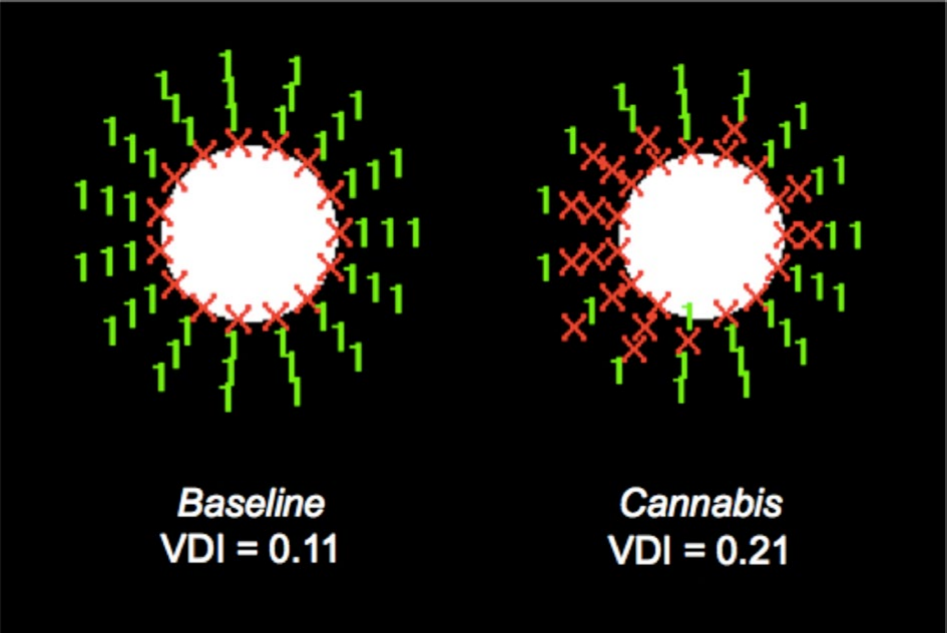
Graph made with the Halo v1.0 software for one participant in the baseline session and after smoking cannabis. VDI value is included. (Pupil size was 5 mm for both sessions).

The intraocular straylight also increased significantly (approximately 9%) after cannabis use (Table 1). As a consequence, the participants perceived more halos, resulting in higher VDIs. Figure 2 shows the graphs pertaining to the Halo software for one participant at the baseline session and after smoking cannabis. After smoking cannabis, the participant presented a greater number of undetected peripheral stimuli (red), resulting in a greater halo area and, therefore, a higher disturbance index. This worsening was found despite the pupil size being the same under the two conditions (5 mm).

As can be seen in Table 1, there were significant differences in the accommodative response, with an increase in accommodative lag (i.e., greater under-accommodation) after smoking cannabis at 0.4 m and 0.2 m. Stereoacuity also deteriorated, being 50.7% worse in near vision and 213.1% in far vision. Finally, cannabis use produced a 5.2% reduction in pupil diameter at scotopic light levels; this change is statistically significant when compared to the baseline condition (*p* < 0.001). At higher lighting levels there was little difference in pupil size in the two conditions. When comparing sexes, we did not observe any statistically significant differences in visual test results after smoking cannabis.

Table 2 indicates the self-perceived changes recorded by the participants with regard to their quality of vision as a consequence of smoking cannabis. The results of the questionnaire indicated that about 68% of the participants considered that smoking cannabis worsened their vision, while about 32% considered it to have no negative effect. Similarly, about 68% of the participants reported that both glare and halos worsened after cannabis use. Finally, 74% of the respondents felt that smoking cannabis affected their night vision, diminishing their ability to drive at night.

**Table 2:** Participants’ self-perceived changes in visual quality after using cannabis.

	Much worse	Slightly worse	No change	Improved
Do you think that smoking cannabis affects your vision?	3.2	64.5	32.3	0.0
How does cannabis use affect perceived glare?	16.1	51.6	32.3	0.0
How does cannabis use affect halos?	17.3	51.7	31.0	0.0
Do you think that cannabis use affects your night vision, diminishing your ability to drive at night?	16.1	58.1	25.8	0.0

The data is expressed in percentages.

**Figure 3:**
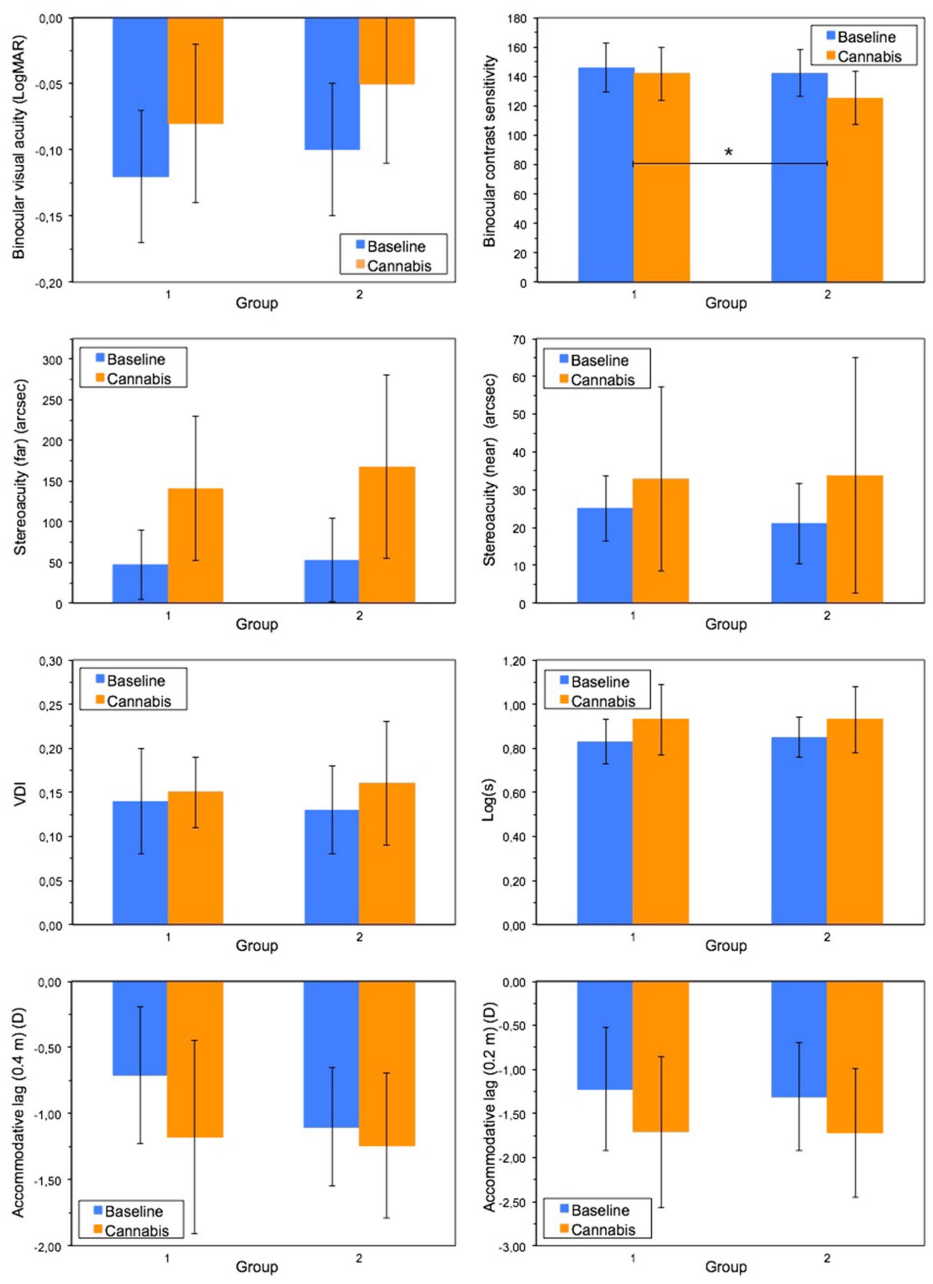
Group comparison for the different visual parameters in the baseline session and after smoking cannabis. * Indicates that the decline after smoking was significantly higher in group 2 than group 1.

In order to evaluate any association between the questionnaire responses to the objective parameters measured in the visual function assessment, the sample was divided into two groups comprising those participants who reported that, in general, they did not perceive their vision to be worse after cannabis use (group 1) and those who, in contrast, said that they did perceive it to be worse (group 2). The first group is thus composed of the participants who indicated in the questionnaire that their vision after cannabis smoking "does not get worse at all" or "gets better", while the second group is made up of those participants who answered that their vision is "much worse" or "a little worse". Table 3 provides the demographic data and mean differences between the conditions (baseline-cannabis) for each visual parameter for the two groups. The average AUDIT and CUDIT-r scores indicated that there was no significant difference in the use frequency/profile, although the group who thought that smoking cannabis did not affect their vision (group 1) did use the substance somewhat more frequently. Both groups also reported almost identical ages when they started consuming and have used cannabis for a similar length of time. The mean differences between the conditions show, firstly, that all variables worsened after cannabis smoking in both groups (Table 3). On the other hand, the comparison between groups (t-test or Mann Whitney U test) reflected the fact that the subjects who thought their vision was worse after smoking cannabis (group 2), showed significantly greater deterioration in contrast sensitivity after use (Fig. 3). This group also presented a greater deterioration in stereoacuity and the visual disturbance index (VDI) than group 1, although these differences were not statistically significant (Fig. 3). In contrast, smoking cannabis supposed a greater increase in straylight level and more accommodative lag, especially at the 40 cm viewing distance, for group 1, but again, the differences were not significant (*p* > 0.05) (Fig. 3).

**Table 3:** Demographic data and mean differences between conditions (baseline-smoking cannabis) for each visual parameter for the two groups classified according their subjective perception of how cannabis affects their vision.

	Group 1 (N = 10) (mean (SD))	Group 2 (N = 21) (mean (SD))	t/z	*p* value
**Demographic data**				
Sex (n, (%))^a^	Male 6 (19.35%) Female 4 (12.90%)	Male 15 (48.39%) Female 6 (19.35%)	− 0.626	0.53
Age (years)	21.20 (1.03)	24.48 (5.94)	2.452	0.022
Monthly use (number of days)	13.75 (9.81)	8.92 (10.96)	− 1.185	0.25
Duration of use (years)^a^	4.20 (2.53)	6.57 (5.65)	− 1.381	0.173
Age when started use (years)	17.00 (2.00)	17.10 (1.92)	0.127	0.900
AUDIT score	6.30 (2.45)	7.52 (3.92)	0.902	0.37
CUDIT-r score	9.90 (3.75)	8.33 (6.25)	0.395	0.40
**Mean differences between conditions**				
Visual acuity^b^ (logMAR)	− 0.05 (0.03)	− 0.05 (0.05)	− 0.405	0.69
Contrast sensitivity^c^	4.23 (13.22)	17.07 (16.13)	2.186	0.029
Stereoacuity (far)^b^ (arcsec)	− 94.00 (85.53)	− 115.00 (96.21)	− 0.584	0.56
Stereoacuity (near)^b^ (arcsec)	− 9.78 (20.11)	− 12.74 (25.96)	− 0.304	0.76
VDI^b^	− 0.01 (0.04)	− 0.03 (0.05)	− 1.150	0.26
Log(s)^b^	− 0.11 (0.13)	− 0.09 (0.12)	0.417	0.68
Accommodative response (lag) (40 cm)^c^ (D)	0.47 (0.49)	0.13 (0.36)	− 2.006	0.056
Accommodative response (lag) (20 cm)^c^ (D)	0.49 (0.56)	0.43 (0.62)	− 0.263	0.80

^a^Mann–Whitney U test.

^b^Negative value indicates worsening after smoking cannabis.

^c^Positive value indicates worsening after smoking cannabis.

The binary logistic regression model showed that, in fact, the deterioration in contrast sensitivity after smoking cannabis is the only significant predictor of a participant’s subjective response to the questionnaire. Thus, the odds ratio of this parameter indicates that the greater the deterioration in contrast sensitivity, the greater the probability of belonging to group 2, and therefore of perceiving a negative effect on vision after smoking cannabis (Odds Ratio 1.066; 95% CI 1.000, 1.137; *p* = 0.049). However, the demographic data (age, sex, cannabis use frequency, AUDIT/CUDIT-r scores) did not present a significant role in the subjective perception of visual changes.

**Figure 4:**
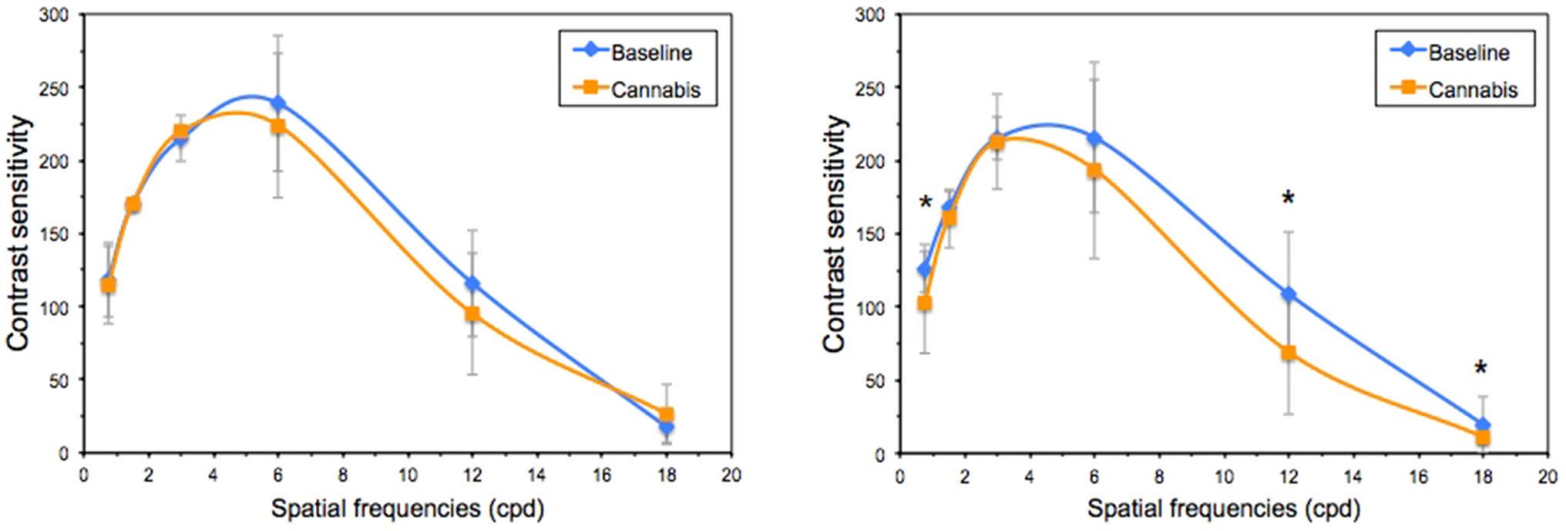
Mean binocular contrast sensitivity function for groups 1 (left) and 2 (right) in the baseline session and after smoking cannabis. * indicates spatial frequencies for which there are significant differences (Wilcoxon test) and error bars indicate the SD. cpd, cycles per degree.

Since contrast sensitivity proved to be the only parameter for which the change after cannabis smoking was significantly associated with the participants' self-perceived visual quality, the next step was to analyze whether the different spatial frequencies had the same influence. The use of this substance did not alter contrast sensitivity in group 1, which showed no significant differences for any of the spatial frequencies studied (*p* > 0.05). In fact, for 1.5 cpd it was exactly the same in both conditions, and even slightly improved in the session involving use for 3 and 18 cpd (Fig. 4). However, for group 2, the contrast sensitivity worsened significantly after smoking cannabis for the spatial frequencies 0.75 cpd (z = − 2.565; *p* = 0.010), 12 cpd (z = − 2.729; *p* = 0.006) and 18 cpd (z = − 2.110; *p* = 0.035) (Fig. 4). Table 4 shows the mean differences between conditions (baseline—smoking cannabis) for the different spatial frequencies and for the two groups studied above. The Mann–Whitney U test indicated a statistically significant difference only for the mean difference in the 18 cpd spatial frequency, for which group 2 experienced significantly greater worsening after cannabis use (Table 4). Finally, the binary logistic regression model including the different spatial frequencies evaluated as predictors indicated that 18 cpd was the only spatial frequency that significantly predicted the subjective perception of the effect of cannabis use on vision (Odds Ratio 1.135; 95% CI 1.006, 1.280; *p* = 0.040).

**Table 4:** Mean differences between contrast sensitivity conditions for all spatial frequencies.

Spatial frequencies (cpd)	Group 1 (N = 10) (mean (SD))	Group 2 (N = 21) (mean (SD))	z	*p* value
0.75	3.80 (12.02)	23.00 (33.62)	− 1.666	0.19
1.5	0.00 (0.00)	7.14 (17.93)	− 1.237	0.55
3	− 5.00 (15.81)	2.14 (36.90)	− 0.307	0.85
6	15.00 (47.43)	21.90 (65.64)	− 0.203	0.85
12	20.80 (34.93)	39.76 (52.28)	− 1.240	0.23
18	− 9.20 (15.54)	8.48 (19.14)	− 2.390	0.017

A positive value corresponds to worsened contrast sensitivity after smoking cannabis.

Mann–Whitney U test was applied for comparisons.

## Discussion

In this study, we evaluated the effects of smoking cannabis on different visual parameters. The results showed that smoking a cannabis cigarette produces a significant deterioration in static visual acuity. Although this visual parameter is one of the most used in studies of this type, the results obtained so far are not conclusive. Adams et al.^42^ found that cannabis use had no effect on static visual acuity for two different levels of contrast (12 and 49%). However, marijuana use produced significant dose-related reductions in dynamic visual acuity^13^, which could be due to impaired ocular motility^21^. Similarly to our result, Noyes et al.^43^ found that taking a 20 mg dose of THC as a pain reliever produced blurred vision in cancer patients. For chronic users who had used the drug for 10 or more years, Dawson et al.^14^ found an optically uncorrectable acuity deficit. The lack of homogeneity in the methods used in studies on this subject could justify the differences between the results, since both the dose and the time elapsed until measurement after administration, as well as the test used, may be influential.

Although visual acuity is the most widely used test to assess the state of visual function, there are other metrics that have been demonstrated to be more important for certain everyday tasks, for example, driving or reading. One of these is contrast sensitivity^44^, and our results have shown that this parameter is significantly worse after smoking cannabis. Lalanne et al.^18^ revealed that CS is permanently altered in cannabis users with an early onset, especially for lower frequencies. Other work has found a reduction in CS in abstaining cannabis users in low light^19^, but not in higher light conditions^45^.

Cannabis use also significantly affected the participants' three-dimensional vision, as we found a significant deterioration of stereoacuity at the two distances evaluated. A number of studies have shown changes in three-dimensional perception caused by cannabis use due to so-called binocular depth inversion illusion. The occurrence of this illusion is reduced both under the effects of cannabis^15,46^, and permanently in regular users^16^. Although the mechanisms of this effect are not known, it seems to be related to a deterioration of visual processing. These changes may be due to cannabis’ influence on the different stages of the visual pathway that contain cannabinoid receptors, such as the thalamus, the lateral geniculate nucleus, and the visual cortex^10,47,48^, thereby altering the process of perception^48,49^. The same issue could be responsible for the changes found in stereoacuity, which negatively impacts an individual’s ability to perform various tasks, and can lead to increased difficulties interacting with the world^50^. One such interaction is driving, in which visual function plays an important role. Cannabis is one of the most prevalent illicit drugs involved in road traffic fatalities, and its negative influence on driving skills have already been reported^29,30,51^. Driving is highly dependent on vision, and the changes observed in this study imply a significant increase in risk.

In addition to stereopsis, another determining visual function for tasks that involve working continuously at different viewing distances is accommodation. An earlier study showed that cannabis users reported reading difficulties^21^, which could be related to the effects of this drug on accommodation. Although two publications mention reduced accommodation in cannabis users^52,53^, to the best of our knowledge, there are no experimental studies fully assessing the effects of this drug on accommodation. Our results show that cannabis use induces an increase in accommodative lag, which could be due to the interaction of this substance with the CB1 cannabinoid receptors located in the visual system. Some of these receptors are situated in the ciliary muscle, which could affect the accommodation process^54^, and therefore, the accommodation response.

Nighttime vision can also be affected by cannabis use, as demonstrated by the visual disturbance index and the level of straylight. Our results indicate that a person who has smoked cannabis is more sensitive to glare, which is manifested as a greater halo extension that impairs their ability to discriminate stimuli around light sources. Contrary to our findings, some case studies state that cannabis has a positive effect on night vision, improving dark adaptation and scotopic sensitivity^22,23^. The different results may be due to the smaller sample sizes in the studies that observed an improvement in night vision, but results may also depend on the dose and/or type of cannabis consumed by subjects. Conversely, other work has shown results more in line with ours. For instance, it has been shown that regular cannabis users need more time to recover from glare^12^ and more time to adapt to darkness^14^. Larger pupil size may be responsible for these changes, but current results on the effect of cannabis use on pupil size are contradictory^14,55^. The worsening of night vision quality could then be due to a deterioration of the optical quality of the eye, with the tear film being responsible. Tear secretion has shown to be reduced after smoking cannabis^56^. In addition, cannabis has an anesthetic effect^57^ that reduces corneal sensitivity. This causes less frequent blinking^58^ making the tear film less stable and creating an irregular refracting surface. This circumstance could lead to a worsening of the optical quality of the visual system and could cause an increase in straylight levels, which in turn would lead to more influence of halos and greater glare. Future studies are necessary for a more in depth analysis of these points.

According to the questionnaires we gave to the participants, about two thirds are aware of the negative effect of cannabis on their visual function, although very few indicate that it affects them to any great extent. There is a widespread belief that cannabis is a soft drug and that it even has positive effects at the brain level^26,27^. In terms of vision, a recent study compared self-reported eyesight quality between heavy marijuana smokers with youths who never used marijuana and light marijuana users without finding any statistically significant differences^28^.

In our study, the results obtained from comparisons between the group who thought their vision was worse after smoking cannabis and the group who thought their vision was unchanged showed that of the mean differences between sessions, contrast sensitivity was the only one that differed significantly. We found that this function is objectively worse in those people who subjectively indicate that their vision is worse after using cannabis. Furthermore, this variable is the only significant predictor of a participant's subjective response to the questionnaire on the visual effect of smoking cannabis. In particular, the highest spatial frequency (18 cpd) has the greatest influence on the perception of one's visual state. The visual environment is full of stimuli with different levels of contrast, so to properly perform tasks in our daily lives, this visual function is critically important^44,59^.

Even though in this study we found that cannabis use had a significant effect on the visual function of occasional users, we must consider the methodological limitations of interpreting such results. On the one hand, the fact that each participant smoked the cannabis cigarette following their normal consumption pattern does not allow us to establish a relationship between dose or the type of cannabis used and its effect. Therefore, the effects of cannabis in relation to the specific compounds inhaled (e.g., THC and cannabidiol) could also be investigated in future studies. Furthermore, by not measuring the concentration in the blood, we cannot relate this to the effect on the various tests. However, the relationship between dose, blood concentration and effect is not linear^41^. Our aim was to study the consequences that habitual use, which the participants may carry out on a normal day, has on vision, and these results can serve as a starting point for future work involving different doses, or different administration pathways. It would also be of interest to include some electrophysiological, imaging, or pharmacological tests in order to explain the changes in the signaling along the visual pathway. This would allow us to form associations between the functional effects noted in this study and the neuronal or signaling effects. On the other hand, our sample size limited the number of categories we could establish to assess the participants’ subjective perception of cannabis’ effects. With a larger sample and more variability in terms of the consumption profile we could probably have established different categories of perceived effects (e.g., “slightly impaired”, “very impaired”, “no difference”, and “improves”). So we believe future research is needed in order to explore the effects of cannabis on visual performance, but also to examine the relationship between (objectively evaluated) visual effects and the subjective perception of such effects. Finally, although previous findings suggest that cannabis may have a different effect on males and females^60,61^, we did not observe such a difference. This could be because of the sample size and different distribution of males and females. Future studies with larger samples are also needed to explore whether males and females experience different visual effects.

In summary, this study shows that smoking cannabis has significant adverse effects on certain visual functions, including visual acuity and contrast sensitivity, as well as in nighttime-related visual parameters such as the VDI and intraocular straylight. These latter functions have been evaluated for the first time while the participants were under the influence of cannabis. Our study also examined the relationship between objective changes in visual parameters after smoking cannabis and the relationship with the users' own subjective perception, identifying contrast sensitivity as the only visual function significantly associated with the participants’ responses. Our results could help generate a better understanding of the visual changes related to cannabis use and their implications for everyday tasks, raising awareness among users of the risks involved consuming this drug.

## Data Availability

The datasets generated during the current study are available from the corresponding author on reasonable request.
